# Whole Genome Sequencing for the Analysis of Drug Resistant Strains of *Mycobacterium tuberculosis*: A Systematic Review for Bedaquiline and Delamanid

**DOI:** 10.3390/antibiotics9030133

**Published:** 2020-03-23

**Authors:** Luisa Maria Nieto Ramirez, Karina Quintero Vargas, Gustavo Diaz

**Affiliations:** 1 Facultad de Ciencias Básicas, Universidad Santiago de Cali, Cali 760035, Colombia; 2 Facultad de Ciencias para la Salud, Departamento de Ciencias Básicas, Universidad de Caldas, Manizales 170002, Colombia; karinaquinterovargas@gmail.com; 3 Centro Internacional de Entrenamiento e Investigaciones Médicas (CIDEIM), Cali 760031, Colombia; diaz.gustavo2011@gmail.com; 4 Facultad de Ciencias Naturales, Universidad Icesi, Calle 18 No. 122-135, Cali 760031, Colombia

**Keywords:** Drug resistance, WGS, SNP, mutations, bacteria, clinical isolates

## Abstract

Tuberculosis (TB) remains the deadliest Infectious disease worldwide, partially due to the increasing dissemination of multidrug and extensively drug-resistant (MDR/XDR) strains. Drug regimens containing the new anti-TB drugs bedaquiline (BDQ) and delamanid (DLM) appear as a last resort for the treatment of MDR or XDR-TB. Unfortunately, resistant cases to these drugs emerged just one year after their introduction in clinical practice. Early detection of resistant strains to BDQ and DLM is crucial to preserving the effectiveness of these drugs. Here, we present a systematic review aiming to define all available genotypic variants linked to different levels of resistance to BDQ and DLM that have been described through whole genomic sequencing (WGS) and the available drug susceptibility testing methods. During the review, we performed a thorough analysis of 18 articles. BDQ resistance was associated with genetic variants in *Rv0678* and *atpE,* while mutations in *pepQ* were linked to a low-level of resistance for BDQ. For DLM, mutations in the genes *ddn, fgd1, fbiA*, and *fbiC* were found in phenotypically resistant cases, while all the mutations in *fbiB* were reported only in DLM-susceptible strains. Additionally, WGS analysis allowed the detection of heteroresistance to both drugs. In conclusion, we present a comprehensive panel of gene mutations linked to different levels of drug resistance to BDQ and DLM.

## 1. Introduction

Multidrug-resistant tuberculosis (MDR-TB, caused by *Mycobacterium tuberculosis (Mtb)* resistant to isoniazid and rifampicin) is a matter of deep concern worldwide, causing more than 200,000 deaths yearly [1]. It was estimated that half a million people developed MDR-TB in 2018; however, only 190,000 cases were reported to the World Health Organization (WHO) in 2018, which represents a 62% MDR-TB detection gap [1]. A more severe form of drug resistance is extensively drug-resistant TB (XDR-TB, defined as MDR-TB with further resistance to at least one fluoroquinolone and one injectable agent). XDR-TB caused around 14,000 cases in 2018 [1]; nonetheless, the actual prevalence of this resistant form of TB is unknown.

**Figure 1:**
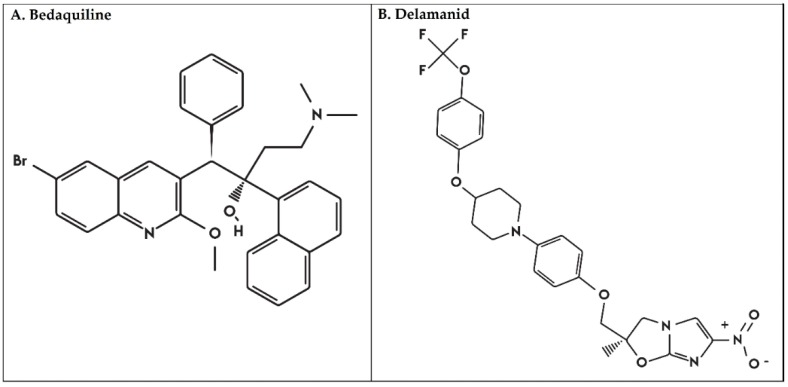
Chemical structure of (**A**). Bedaquiline and (**B**) Delamanid.

Recently, two new drugs were approved for the treatment of drug-resistant TB and are currently being tested in shorter regimens [2]. Bedaquiline (BDQ) (formerly called TMC207 or R207910) was developed by Janssen Pharmaceuticals in 2005. BDQ is a diarylquinoline (Figure 1A) that inhibits an ATP synthase of *Mtb* and was first endorsed by the WHO for the treatment of MDR-TB in 2013 [2]. The second drug is delamanid (DLM), a dihydro-nitroimidazooxazole derivative (former OPC-67683, Figure 1B) developed by Otsuka Pharmaceutical in 2003. DLM inhibits the synthesis of cell wall mycolic acids [3]. DLM was recommended for the treatment of adults with MDR-TB by the WHO in 2014 and has shown low interaction with antiretroviral therapy [4]. The emergence and spread of drug-resistant *Mtb* to these new drugs, particularly among MDR/XDR TB strains, will impose new obstacles that will threaten global TB control. Unfortunately, the first case of resistance to both BDQ and DLM drugs was reported from a Tibetan TB patient in 2014 [5,6]. 

Drug resistance (DR) can be determined either by phenotypic or genotypic methods. Currently, phenotypic resistance to BDQ and DLM is determined using provisional critical concentration values defined by the WHO or the European Committee on Antimicrobial Susceptibility Testing (EUCAST) [7,8]. However, the established thresholds for BDQ or DLM resistance are highly variable and influenced by the type of culture system used [7]. Regarding molecular makers of resistance, current data suggest that BDQ resistance is associated with mutations in the drug target *atpE*, which encodes for the subunit c of the ATP synthase complex. Additionally, off-target mutations at *Rv0678, pepQ (Rv2535c),* and *Rv1979c* are linked to BDQ resistance [9]. Similarly, off-target mutations at *ddn (Rv3547), fgd1 (Rv0407), fbiA (Rv3261), fbiB (Rv3262),* and *fbiC (Rv1173)* were associated to DLM resistance [6,10,11]. Resistance mechanisms for BDQ and DLM can be found in previous reviews [11,12,13].

Genotypic methods to detect resistance to anti-TB drugs are mostly based on the identification of already stablished genetic markers using PCR-based Sanger sequencing or commercially available tests [14,15]. However, these tests are limited to the most frequently used first- and second-line anti-TB drugs and no such test is available for BDQ or DLM resistance. Alternatively, whole genome sequencing (WGS) has emerged as a powerful method for the study of evolution, pathogenesis, transmission, drug targets, and resistance in *Mtb* [16,17,18]. In fact, WGS is already in place for TB surveillance in some European countries, focusing on populations at a higher risk of DR-TB [19]. To our knowledge, there are no systematic reviews about the specific application of WGS in *Mtb* to predict BDQ and DLM resistance. Here, we aimed to describe the bacterial genetic variations exclusively identified by WGS that were associated with BDQ or DLM resistance in *Mtb*, comparing the genetic variations observed with the phenotypic drug susceptibility profile.

## 2. Results and Discussion 

### 2.1. Description of Collected Articles

**Figure 2:**
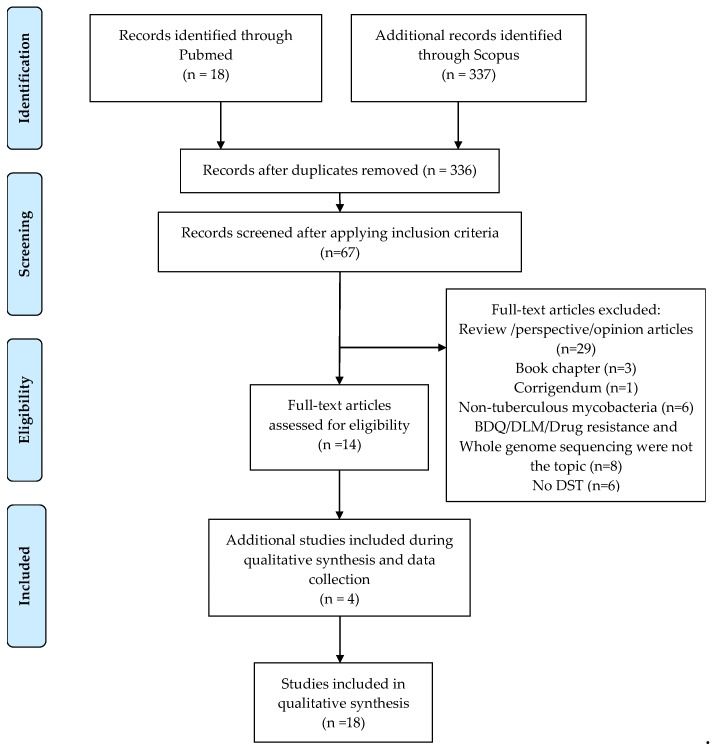
The systematic review process using Preferred Reporting Items for Systematic Reviews and Meta-Analyses (PRISMA) guidelines [20].

In the initial search, 337 published studies were found after merging duplicates. Later, we applied the inclusion and exclusion criteria, selecting 14 articles for qualitative analysis. We added four publications during the reading and synthesis step, giving us a total of 18 articles selected for this review. The workflow was summarized using the Preferred Reporting Items for Systematic Reviews and Meta-Analyses (PRISMA) diagram (Figure 2) [20]. Analyzed variables are described in Tables S1–S3. Most of the studies reported resistance to BDQ (17/18, 94.4%), compared to DLM (6/18, 33.3%). The initial reports of BDQ and DLM resistance through WGS were published in 2014 and 2015, respectively (Table S1) [6,21], one year after these drugs were endorsed for the treatment of MDR-TB patients [4,22]. Ten studies included *Mtb* strains from MDR/XDR-TB patients. The number of patients in these studies ranged from 1-517 (Table S1). Seven studies evaluated laboratory strains (H37Rv and ATCC strains such as ATCC35822, among others), including in vitro induced or spontaneous mutants after exposure to BDQ [23], and strains that developed BDQ resistance in the mouse model [24,25].

Eight publications presented longitudinal studies where BDQ or DLM resistance was developed after (in vivo or in vitro) drug exposure [5,6,25,26,27,28,29,30]. Some of these longitudinal studies evaluated two to eight isolates from the same patient. In one case, DLM resistance arose as soon as three months after drug administration, emphasizing the need to evaluate combined drug-therapies while using DLM to prevent the early development of DLM-resistant strains. These findings are relevant since DLM, as well as BDQ, are recommended for MDR/XDR TB patients, where limited treatment options are left. 

### 2.2. Phenotypic Methods for Drug Susceptibility Testing (DST) for BDQ and DLM

**Figure 3:**
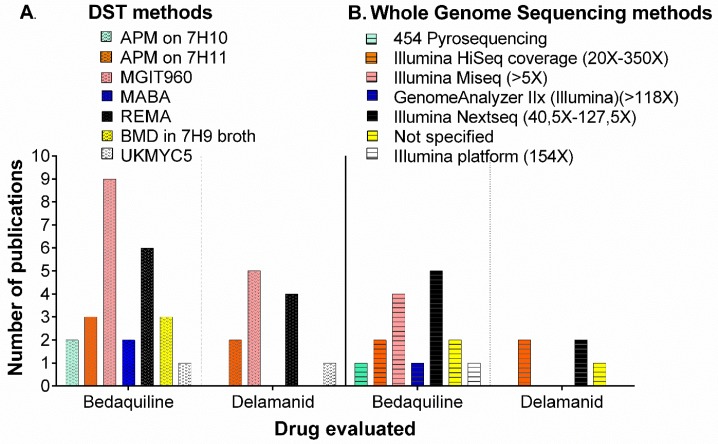
Methods used in the evaluated studies for (**A**). Drug susceptibility testing (DST) and (**B**). Whole genome sequencing methods. Coverage in parenthesis when available. APM: Agar proportion method, MABA: Microplate alamarBlue Assay, REMA: Resazurin microtiter assay, BMD: Broth Microdilution, UKMYC5: microdilution plate includes two new (bedaquiline and delamanid) and two repurposed (clofazimine and linezolid) compounds. NS: Not specified.

More than half of the studies performed two or more phenotypic methods to evaluate drug resistance (10/18, 55.6%). MGIT960 was the most frequently used DST method for both drugs (Figure 3A, Table 1). The broth microdilution (BMD) test was used in three formats: resazurin microtiter assay (REMA), the commercial compound alamarBlue^®^ (ThermoFisher) (MABA), and 7H9 broth (Figure 3A). Interestingly, there was a newly introduced method: the UKMYC5 plate, which is a 96-well microtiter plate designed by the CRyPTIC Consortium [31,32]. The latter method exhibited lower intra- and inter-laboratory reproducibility (<95%) for BDQ compared to DLM [32]. 

The interim critical concentration (CC) for BDQ susceptibility recommended by WHO is 0.25 mg/L when using 7H11 agar proportion method (APM), or 1.0 mg/L when using MGIT960. For DLM, the interim CC is 0.016 mg/L using 7H11 or 0.06 mg/L using MGIT960 [7,33]. In its latest version (Ver. 10 from 01/01/2020), the EUCAST has established a clinical breakpoint (CB) of 0.25 mg/L for BDQ if using Middlebrook 7H11/7H10 medium. For DLM, the EUCAST CB was set at 0.06mg/L [8]. CC is defined as the lowest anti-TB concentration that inhibits >99% of drug-susceptible strains in vitro, while CB is a concentration that separates strains that will likely not respond to treatment [7]. The CC values reported for BDQ and DLM are summarized in Table 1. The mode for the CC for BDQ testing by MGIT960 and APM was the same CC already established by the WHO (Table 2) [24]. For the REMA method, two studies reported a CC of 0.25 mg/L for BDQ, that was also reported when using BMD and MABA (Table 2) [27].

**Table 1:** Critical concentration (CC) or cutoff defined in the evaluated articles. DST: drug susceptibility testing.

DST Method	CC for Bedaquiline (BDQ) (mg/L)	CC for Delamanid (DLM) (mg/L)
(Bactec) MGIT960	0.8 [6] 1.0 [29,30,32,34] 2.0 [35]	0.04 [5,6] 0.06 [30] 0.12-0.125 [10,32]
Resazurin microtiter assay (REMA)	0.125 [27] 0.25 [21,26]	0.03 [30]
Agar proportion method (APM on 7H10 or 7H11)	0.12 [24] 0.25 [23,28,32,36]	0.06 [32] 0.2 [10]
Broth Microdilution (BMD)	0.25 [35,37]	Not defined
Microplate alamarBlue Assay (MABA)	0.25 [37]	Not defined

In all articles included in this review, most of the clinical isolates and laboratory strains of *Mtb* were classified as resistant to BDQ and DLM following either the WHO or EUCAST cut-offs. However, in some instances, the phenotypic methods (7H11 APM and MGIT960) showed discrepant results for BDQ. In one study, two *Mtb* strains were BDQ resistant using MGIT960 (minimum inhibitory concentration (MIC) of 2 mg/L) but susceptible based on the 7H11 APM method (MIC ≤ 0.25 mg/L). Of note, these strains were isolated from two XDR-TB patients who had been treated with BDQ-containing regimens that failed. These patients had BDQ susceptible strains (MIC of 0.03 mg/L) originally. Here, we observed an agreement between the clinical outcome, MGIT960 results, and the MIC increase pre- and post-BDQ exposure. Additionally, WGS data confirmed the BDQ-resistant profile of these strains. 

### 2.3. Mtb Genes Associated with BDQ and DLM Resistance 

For this review, we focused on mutations identified only by next-generation WGS, which are summarized in Table 2. The WGS methods used for evaluating resistance associated mutations (RAM) for each drug are described in Table S1 and Figure 3B. The Illumina NextSeq was the most frequently used platform for both drugs (Figure 3B). A significant advantage of Next Generation Sequencing (NGS) over traditional Sanger sequencing is its deeper coverage [38]. In the articles reviewed here, the coverage ranged from 5X-350X, reaching the highest coverage through Illumina HighSeq4000 [39] (Figure 3B), with higher confidence for detecting genetic variants. Additionally, eight studies confirmed mutations through other genotypic methods (PCR-based Sanger sequencing or targeted deep sequencing) (Table S1).

Previous studies have also identified BDQ RAM at *Rv0678, atpE,* and *pepQ* genes, but we did not include them because they were determined by PCR-Sanger sequencing [9,40,41,42]. The mutations shared between our reviewed studies associated to BDQ resistance (identified by WGS) and studies that applied first-generation sequencing methods are shown in Table 3. Similarly, DLM RAM have been also identified through PCR-Sanger sequencing in other studies [43,44]. However, none of them shared any of the mutations that we describe here in this review. 

#### 2.3.1. Mutations Associated with BDQ Susceptibility

**Figure 4:**
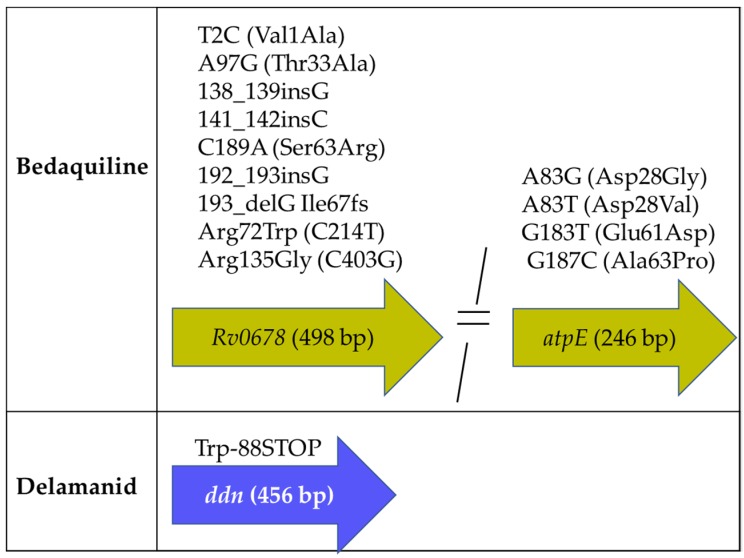
Mutations identified by WGS reported in two or more studies and exclusively present in bedaquiline- and delamanid-resistant strains.

We found seven genes with different types of mutations in BDQ-resistant strains (Table 2). The most frequent mutations were identified at the off-target gene *Rv0678*, followed by mutations in the drug target *atpE* (Table 2 and Figure 4). The most frequently reported variations in *Rv0678* included five non-synonymous Single Nucleotide Polymorphisms SNPs, three insertions, and one deletion, while the most frequent mutations in *atpE* comprised four non-synonymous SNPs (Figure 4). Remarkably, we found three *atpE* Single Nucleotide Variants (SNVs) at the aminoacid position 28 (two of them described in Figure 4 and the remaining in Table S2). Additional mutations at this position (Asp28Asn [41] and Asp28Pro) have been found in *Mtb* clinical strains with increased MIC to BDQ, using PCR-Sanger sequencing [42]. The latter suggests that *atpE* mutations that affect the aminoacid residue 28 could be specific markers for BDQ resistance. Additional mutations found at *pepQ* were associated with different levels of BDQ susceptibility (MICs of 0.12 mg/L, using APM) [24]. 

*Rv0678,* also recognized as *mmpR,* is hypothesized to act as a repressor of the Mmps2–MmpL2, MmpS4–MmpL4 and MmpS5–MmpL5 transporter/transport systems [46]. MmpS–MmpL proteins act as transporters for *Mtb* cell wall lipids, virulence factors, and some drugs. MmpS5–MmpL5, particularly, participates in the export of BDQ, clofazimine, and azoles such as econazole [47]. Mutations in *Rv0678* lead to the inactivation of the MmpS5–MmpL5 repressor, increasing the efflux of BDQ and clofazimine. Furthermore, *atpE* encodes the transmembrane subunit of the ATP synthase complex of *Mtb*, which is known to be the target of BDQ. AtpE was discovered as a BDQ target by mutational and computer-based molecular modeling analyses, highlighting the amino acid reside Glu-61 as the binding site for BDQ [48,49,50]. In our review, one of the mutations was identified at this amino acid position (Figure 4), and that mutation has been also detected by first-generation sequencing approaches (Table 3). According to the Protein–Protein Interaction Networks analysis using STRING (https://string-db.org/), Rv0678 has statistically relevant co-occurrences with the uncharacterized transporter Rv1979c and the cytoplasmic peptidase PepQ (Rv2535c), both encoded by genes associated with cross-resistance between BDQ and clofazimine. 

All *atpB* mutants that exhibited BDQ resistance have, simultaneously, *atpE* RAM [24]. Additionally, there was only one *atpB* mutant without a simultaneous *atpE* mutation that was BDQ sensitive (Table S2), suggesting that mutations at *atpB* are not linked to BDQ resistance per se. Both *atpB* and *atpE* belong to the F_0_ operon, together with *atpF,* which forms the membrane proton channel of the ATP synthase complex [42]. Except for *atpE* mutations, there are no mutations reported in the genes of the F_0_ nor the F_1_ operons in BDQ-resistant strains [42]. It seems like the *atpB* mutations found so far are mostly accessory mutations that probably could compensate the fitness cost that may impose the *atpE* mutation, as previously suggested [51]. This also may have occurred for the *ppsC* mutant (*n* = 1) and *Rv1979c* mutants (5/6 strains) that all had simultaneous mutations at *Rv0678* and were BDQ resistant (Table S2). WGS analysis will help to detect these accessory mutations. 

While acquiring resistance to rifampicin and isoniazid has been demonstrated to modify *Mtb* fitness [52,53,54], mutations in *Rv0678* and *atpE* (or *atpB*) have not been shown to alter the *Mtb* fitness cost in vivo and in vitro, respectively [26,42]. However, the low frequency of *atpE* mutants in the clinical setting make some authors suggest a possible reduced fitness cost linked to some *atpE* mutations [41]. Likewise, *pepQ* and some *mmpL5 Mtb* mutants have shown reduced fitness [24,55]. 

#### 2.3.2. Mutations Associated with DLM Susceptibility

We identified four genes with RAM present in DLM-resistant strains and some of these mutations were also present in DLM susceptible strains (Table 2, Table S3). Among those, *ddn* had a premature stop codon mutation (Trp88STOP**), exclusively found in DLM-resistant strains; while *fbiA* had the mutation Arg175His present in one DLM-resistant and nine susceptible strains (Figure 4, Table S3). Except for *fbiB*, all genes harbored RAM (Table 2). 

So far, the genes associated with DLM resistance are mostly involved in the activation from prodrug to the active form of the drug. For instance, *ddn* (Rv3547), which participates in the activation of DLM and pretomanid, is the deazaflavin (F_420_)-dependent mycobacterial nitroreductase where most of the DLM RAM have been identified [56]. Other mycobacteria species such as *Mycobacterium leprae* lack the *ddn* gene, which make them naturally resistant to DLM and pretomanid [57]. In the same sense, *fbiA (Rv3261), fbiB (Rv3262),* and *fbiC (Rv1173)* are essential for the mycobacterial F_420_ synthesis that together with the glucose-6-phosphate dehydrogenase encoded by *fgd1 (Rv0407)* complete the conversion of DLM to its active state [58,59].

The fitness cost for *ddn* mutants is greatly influenced by the fact that F_420_ plays important roles in *Mtb* physiology and during in vivo growth. F_420_ is known to be essential, especially during hypoxic growth, as well as for protection against redox responses from the host-immune system. If the *ddn* mutation results in the loss of native enzyme activity, a loss in the bacterial fitness is likely. However, some *ddn* mutants have been recovered from MDR-TB patients and mouse lungs, indicating the ability of these mutants to survive under stressful environments. Additionally, the mouse model did not show an impaired growth of specific *ddn* mutants, compared to wild type strains [60]. 

### 2.4. Other Findings: Mutations in Drug-Susceptible Strains, Cross-Resistance, and Heteroresistance

A total of 110 and 11 non-synonymous mutations were found in BDQ- or DLM-susceptible strains, respectively (Table 2, Tables S2 and S3). Some of these mutations were linked to different *Mtb* lineages. For example, the mutations Lys270Met and Lys296Glu at *fbiA*, found in DLM susceptible strains, were associated with the *Mtb* Haarlem and *M. africanum* WA2 lineages, respectively [44]. Furthermore, the −11C >A mutation at *Rv0678* [27] was also found in 44 *Mtb* strains of different genetic lineages, all of them with BDQ MIC values ≤0.03 mg/L [9].

We also identified two important phenomena of clinical interest: cross-resistance and heteroresistance. Some mutations at *Rv1979c*, *pepQ,* and *Rv0678* caused cross-resistance to clofazimine [21,28,34,35]. Additionally, all the genes identified for DLM resistance here (*ddn, fgd1, fbiA*, and *fbiC*) are also recognized molecular markers of pretomanid resistance [11]. Heteroresistance occurs when a subpopulation of apparently isogenic bacteria exhibits drug-resistance within a population of drug-susceptible strains [61]. We identified three studies that reported heteroresistance for BDQ [29,30,36], one for DLM [30], and one to other drugs [6]. WGS is currently the finest approach to detect heteroresistance and, to some extent, can predict the selection of resistant strains within a patient. In this way, the early detection of heteroresistant variants could optimize the treatment for TB patients that will translate into a better clinical outcome. For the future, it will be interesting to evaluate novel combined therapies directed to heteroresistant strains, potentiating the available anti-TB drug options, and determining the efficacy of these novel strategies to prevent selective pressure. This is in line with the idea of personalized treatment regimens where the characterization of *Mtb* strains by WGS could play a crucial role. 

We acknowledge that the main limitation of this review is the limited number of analyzed articles (*n* = 18). This limitation could be explained by the relatively recent endorsement of BDQ and DLM for the treatment of MDR-TB patients and the fact that BDQ is currently authorized only in 62 countries [62], while DLM in 39 countries [63]. Another reason is the difficulty in obtaining these anti-TB compounds for DST [64]. In fact, six articles were excluded from this review because they did not perform DST, despite having WGS data showing BDQ and DLM RAM [64,65,66,67,68,69].

## 3. Materials and Methods

### 3.1. Data Collection

We selected two databases, PubMed (National Library of Medicine) and Scopus, to complete the article search, using the terms: *Mycobacterium tuberculosis*, bedaquiline, delamanid, resistance, and whole-genome sequencing. These words were used in combination with the Boolean operators “AND” and “OR” in “all fields” without applying additional filters.

The PubMed resulting algorithm search was ((“mycobacterium tuberculosis”[MeSH Terms] OR (“mycobacterium”[All Fields] AND “tuberculosis”[All Fields]) OR “mycobacterium tuberculosis”[All Fields]) AND ((“OPC-67683”[Supplementary Concept] OR “OPC-67683”[All Fields] OR “delamanid”[All Fields]) OR (“bedaquiline”[Supplementary Concept] OR “bedaquiline”[All Fields]))) AND (“whole genome sequencing”[MeSH Terms] OR (“whole”[All Fields] AND “genome”[All Fields] AND “sequencing”[All Fields]) OR “whole genome sequencing”[All Fields]). While the Scopus search algorithm was (whole AND genome AND sequencing) AND (delamanid OR bedaquiline) AND (mycobacterium AND tuberculosis).

### 3.2. Data Items and Quality Assessment

Three researchers independently applied both searching algorithms to ensure the consistency in the number of retrieved articles at each database. On December 12, 2019, the search was repeated to include relevant items for the topic, obtaining a total of 355 studies. Duplicates were merged using Zotero (https://www.zotero.org/), giving a total of 336 publications. The analysis, critical reading, and the quality of the studies was carried out according to the Preferred Reporting Items for Systematic Reviews and Meta-Analyses (PRISMA) [15], which is summarized in Figure 1.

### 3.3. Inclusion and Exclusion Criteria

After the duplicates were merged, the resulting articles were unified in EndNoteX9 and Microsoft Excel, to continue with the selection of inclusion and exclusion criteria. Articles that had the terms: “Delamanid,” or its synonyms, “OPC-67683”, “OPC67683”, “Deltyba”; “Bedaquiline” or its synonyms “Sirturo,” “TMC207”, “R207910”, “AIDS222089” and “variation,” either in the title or abstract were included for further review. We excluded full-text articles that were not research articles (such as reviews, opinions, book chapters, among others), or research articles that did not perform WGS in *Mtb* nor DST for BDQ and/or DLM. An additional four relevant studies for this review were included during the reading and qualitative analysis process (Figure 1). 

### 3.4. Data Extraction Process

From the resulting articles, the following information/variables were extracted: 1. Descriptive analysis of the studies (such as year of publication, evaluated drug, number of clinical and/or laboratory strains assessed, number of longitudinal studies, and isolates that develop DR after drug exposure), 2. Technical details of the phenotypic methods for drug susceptibility (including minimum inhibitory concentration (MIC) reported for each drug and number of resistant isolates by DST), and finally, 3.Technical details regarding genetic WGS (platform or methodology used, genome coverage, number of genes and types of mutations published, the number of mutated strains, and other genotypic tests used).

## 4. Conclusions 

This work provides a summarized panel of mutations linked to DLM and BDQ resistance detected through WGS. DLM and BDQ are currently distributed in a limited number of countries [62,63]; however, RAM in BDQ naïve *Mtb* strains have been already identified [30,35,37]. The latter suggests that BDQ/DLM *Mtb*-resistant strains could be circulating even in countries where these drugs have not been introduced yet. Therefore, it is crucial to have efficient strategies such as WGS to detect BDQ/DLM-resistant strains, anticipating the efficacy of BDQ and DLM for the treatment of MDR/XDR TB. 

The integration of WGS methodologies with DST as part of the routine diagnosis of TB patients (before and after drug treatment) will allow the discovery of RAM, as well as heteroresistance in a cleaner way. This is relevant in the context of the increasing number of DR cases and the need to detect them early, which is still a challenge for TB (62% MDR-TB detection gap) [1]. Considering the absence of definitive cut-off values to define DLM or BDQ resistance, the MIC increase in sequential strains from the same patient should be evaluated. Increasing MIC values would suggest the emergence of drug-resistant strains, as demonstrated in the longitudinal studies reviewed here. In these cases, WGS would be crucial for discovering and confirming new drug-resistance associated mutations. For the future, organizations such as the WHO and EUCAST should work together in order to define standard protocols to detect BDQ and DLM resistance, provide standard (or reference) antibiotic compounds and drug-resistant reference *Mtb* strains, and provide external quality assurance. This could help to unify the CC and CB criteria that are specifically needed for DLM. 

## Figures and Tables

**Table 2:** Non-synonymous mutations classified according to the drug susceptibility profile of the strains.

Genes Linked to BDQ Susceptibility	Total Mutations	R	I ^a^	S	Genes Linked to DLM Susceptibility	Total Mutations	R	S
*Rv0678*	48	39	1	9 ^b^	*fbiA*	6	4	3 ^f^
*atpE*	10	8	0	2	*fgd1*	4	2	2
*Rv1979c*	4	2 ^c^	1	3 ^d^	*ddn*	4	2	2
*pepQ*	101	3	0	98	*fbiC*	3	1	2
*mmpL5*	2	1	0	1	*fbiB*	3	0	3
*atpB*	3	2 ^e^	0	1	−	−	−	
*ppsC*	1	1 ^c^	0	0	−	−	−	

a. Category defined by one article referring to strains with MIC = 2 mg/L using the MGIT 960 method or MIC = 0.25 mg/L using the broth microdilution method [35]. b. One mutation was also present in a BDQ-resistant strain. c. Some of the mutant strains also harbored mutations at *Rv0678.* d. One mutation also identified in resistant and intermediate strains. e. Mutants with *atpE* mutations. f. One mutant also found in a DLM-resistant strain. R: resistant, S: susceptible, I: intermediate.

**Table 3:** Non-synonymous mutations identified through whole genome sequencing (WGS) and described by other PCR-Sanger sequencing studies.

*Gene*	Mutations Identified Through WGS by the Articles Reviewed Here	Studies That also Identified the Mutation by PCR-Sanger Sequencing *
***Bedaquiline***		
***Rv0678 (mmpR)***	−11 C>A	[9]
	T2C (Val1Ala)	Reported as fMet1Ala [45]
	T136C (Cys46Arg)	[41]
	136_137 insG	[41]
	138_139 insG	[41]
	141_142 insC	[41]
	C189A (Ser63Arg)	[40]
	192_193_InsG	[9]
	T350G (Leu117Arg)	[9]
	T407C (Leu136Pro)	[40]
***atpE***	A83T (Asp28Val)	[42]
	G183T (Glu61Asp)	[42]

* These studies did not perform WGS.

## Supplementary Materials

The following are available online at https://www.mdpi.com/2079-6382/9/3/133/s1, Table S1: A general overview of the articles included in this review, Table S2: Non-synonymous mutations associated with the Bedaquiline-susceptibility profile, Table S3: Non-synonymous mutations associated with the Delamanid-susceptibility profile.
